# Synergistic anti-tumor effects of liraglutide with metformin on pancreatic cancer cells

**DOI:** 10.1371/journal.pone.0198938

**Published:** 2018-06-13

**Authors:** Ran Lu, Jin Yang, Rui Wei, Jing Ke, Qing Tian, Fei Yu, Junling Liu, Jingjing Zhang, Tianpei Hong

**Affiliations:** Department of Endocrinology and Metabolism, Peking University Third Hospital, Beijing, China; University of South Alabama Mitchell Cancer Institute, UNITED STATES

## Abstract

Either metformin or liraglutide has been reported to have anti-tumor effects on pancreatic cancer cells. However, it is not clear whether their combined treatment has additive or synergistic anti-tumor effects on pancreatic cancer cells. In this study, the human pancreatic cancer cell line MiaPaca-2 was incubated with liraglutide and/or metformin. The cell Counting Kit-8 (CCK-8), colony formation, flow cytometry, and wound-healing and transwell migration assays were used to detect cell viability, clonogenic survival, cell cycle and cell migration, respectively. RT-PCR and western blot analyses were used to determine the mRNA and protein levels of related molecules. Results showed that combination treatment with liraglutide (100 nmol/L) and metformin (0.75 mmol/L) significantly decreased cell viability and colony formation, caused cell cycle arrest, upregulated the level of pro-apoptotic proteins Bax and cleaved caspase-3, and inhibited cell migration in the cells, although their single treatment did not exhibit such effects. Combination index value for cell viability indicated a synergistic interaction of liraglutide and metformin. Moreover, the combined treatment with liraglutide and metformin could activate the phosphorylation of AMP-activated protein kinase (AMPK) more potently than their single treatment in the cells. These results suggest that liraglutide in combination with metformin has a synergistic anti-tumor effect on the pancreatic cancer cells, which may be at least partly due to activation of AMPK signaling. Our study provides new insights into the treatment of patients with type 2 diabetes and pancreatic cancer.

## Introduction

Pancreatic cancer is the tenth most prominent type of malignant tumor in humans, with a low rate of early diagnosis, high malignancy, and a five-year-survival rate of only 6% [1]. Based on several clinical studies and meta-analysis, it is well accepted that diabetes is one of the risk factors for pancreatic cancer [2]. Patients with diabetes show about a 2-fold risk of developing pancreatic ductal adenocarcinoma (PDAC) [2,3]. On the other hand, the tumor-derived influence on glucose metabolism can cause the dysfunction of pancreatic beta cells, elevation of blood glucose, and eventually development of diabetes [4]. The prevalence of diabetes in patients with pancreatic cancer ranges from 40% to 64%, and approximately 25% to 50% of those patients have developed diabetes between 6 months and 36 months before cancer diagnosis [2,5]. Due to the high coexisting rate of diabetes and pancreatic cancer in patients, it is of great importance to discover the beneficial effects of anti-diabetic drugs on pancreatic cancer to help clinicians choose better treatments for both diabetes and cancer.

In recent years, cumulative evidence from both clinical and basic studies has shown that the first-line anti-diabetic agent metformin may have anti-tumor effects. Therefore, there are several ongoing clinical trials testing the efficacy and safety of using metformin as an add-on therapy to chemotherapy in patients with pancreatic cancer [6]. By contrast, association between the risk of pancreatic cancer and the use of glucagon-like peptide-1 (GLP-1)-based therapies (including GLP-1 receptor agonists and dipeptidyl peptidase-4 inhibitors) in patients with type 2 diabetes is still under discussion. Earlier animal studies and case-control human studies based on healthcare database or histopathological data of donated human pancreata suggested that GLP-1-based therapies might increase the risks of pancreatitis and pancreatic cancer [7–9]. However, recently published randomized controlled cardiovascular outcome trials with longer follow-up duration and better design did not show any significantly increased risk of either pancreatitis or pancreatic cancer in patients with type 2 diabetes who received GLP-1-based therapies [10,11]. Surprisingly, our previous studies revealed that higher level of GLP-1 receptor in PDAC tissue was associated with better prognosis in patients with PDAC after surgery, and that the GLP-1 receptor agonist liraglutide had an anti-tumor effect on human pancreatic cancer cells both *in vitro* and *in vivo* [12,13]. It is noteworthy that liraglutide is one of the most commonly used GLP-1 receptor agonists in clinical practice and shows cardiovascular protective effect in the cardiovascular outcome trial LEADER study [10]. Besides, the dual therapy of metformin with liraglutide has been recommended as an effective and safe treatment strategy for type 2 diabetes by international influential guidelines [14,15].

Since this dual therapy has been widely used in treating patients with type 2 diabetes and either metformin or liraglutide has anti-tumor effect on human pancreatic cancer cells, it is highly interesting to explore the combined effect of these two drugs on the pancreatic cancer cells. Furthermore, our previous study revealed that metformin and liraglutide had synergistic protective effects on endothelial function by interacting with one another’s signaling pathway [16], which increases the possibility that they may have synergistic anti-tumor effect on the pancreatic cancer cells. Therefore, the aim of this study was to investigate whether the combined treatment of liraglutide and metformin had a synergistic effect on tumor cell growth and migration in human pancreatic cancer cells *in vitro*, and if so, to determine which signaling pathways were involved in this synergistic effect. Our study may provide new evidence and strategy for clinicians when dealing with patients with type 2 diabetes and pancreatic cancer.

## Materials and methods

### Reagents

Liraglutide and metformin were obtained from Novo Nordisk (Bagsvaerd, Denmark) and Sigma-Aldrich (St. Louis, MO), respectively. Anti-Bax, anti-proliferating cell nuclear antigen (PCNA), anti-AMP-activated protein kinase (AMPK) and anti-phosphorylated AMPK (p-AMPK) antibodies came from Cell Signaling Technology (Beverly, MA). Anti-cleaved caspase-3 antibody was purchased from Sigma-Aldrich. Mouse anti-GLP-1 receptor monoclonal antibody was from DSHB (Iowa, IA). Anti-GAPDH antibody was bought from ZSGB-BIO (Beijing, China). IRDye 800CW-conjuaged goat anti-rabbit and goat anti-mouse IgGs were purchased from LI-COR Biosciences (Lincoln, NE).

### Cells and culture conditions

The human pancreatic cancer cell line MiaPaca-2 was purchased from American Type Culture Collection (Manassas, VA). Cells were thawed and passaged 2–3 times to obtain a good status as recommended in DMEM (Life Technologies, Carlsbad, CA) containing 25 mmol/L of glucose supplemented with 10% FBS (Life Technologies) at 37°C and 5% CO_2_ in a humidified incubator. Cells were then cultured in the low-glucose (4.5 mmol/L) DMEM with 10% FBS (complete medium) for 2 passages for adaptation, followed by serum-starved pretreatment for 24 h and subsequent exposure to different pharmacological intervention conditions with 1% FBS for additional periods according to the corresponding studies. All of the experiments were performed with early passage cells (≤ 8 passages) in low-glucose DMEM.

### Cell viability determination

MiaPaca-2 cells were seeded at a density of 5×10^3^/well in 96-well plates with serum-free medium. After pretreatment for 24 h, cells were incubated with liraglutide (10, 100 and 1,000 nmol/L) or metformin (0.25, 0.5, 0.75 and 1.0 mmol/L) in DMEM containing 1% FBS for 48 h. As for the combination treatment experiment, cells were incubated with liraglutide (100 nmol/L) or metformin (0.75 mmol/L) alone or in combination. Cell viability and number was determined by using the Cell Counting Kit-8 (CCK-8; Dojindo Molecular Technologies, Kumamoto, Japan) according to the manufacturer’s instructions.

### Colony formation assay

MiaPaca-2 cells were trypsinized and filtered into single cells, and plated onto 6-well plates at a density of 3×10^3^/well with liraglutide (100 nmol/L) or metformin (0.75 mmol/L) alone or in combination in DMEM containing 1% FBS for 8 days [13]. Colonies were fixed with formalin and stained with crystal violet (Sigma-Aldrich) and colony numbers were counted manually under a microscope.

### Cell cycle determination

MiaPaca-2 cells were seeded at a density of 1.0×10^5^/dish (100 mm) and cultured overnight for attachment. The cells were serum-starved for 24 h and then treated with liraglutide (100 nmol/L) and/or metformin (0.75 mmol/L) for 96 h in DMEM containing 1% FBS. At the end of treatment, cells were harvested, washed with PBS, and fixed with 70% ethanol overnight at -20°C. Cells were subsequently stained with 50 μg/mL of propidium iodide (PI) and 20 μg/mL of RNase for 30 min at 37°C in the dark and subjected to analysis on a flow cytometer (FACSAria II Special Order System, BD Biosciences, San Joe, CA).

### Wound healing assay

MiaPaca-2 cells were plated on a 6-well plate at a density of 1×10^6^/well. After cells grew to 80%-90% confluence, scratching wounds were generated by a 20-μL sterile pipette tip. Cells were washed with PBS and then cultured with liraglutide (100 nmol/L) and/or metformin (0.75 mmol/L) in DMEM containing 1% FBS. Photographs were taken at 0 and 48 h after wound generation under a microscope (200×). The relative migration distance was calculated as: (mean wound width–mean remaining width)/mean wound width.

### Transwell migration assay

The assay was performed in transwell chambers (24-well insert, pore size of 8 μm; BD Biosciences). We suspended 2×10^4^ MiaPaca-2 cells in serum-free medium and seeded into the upper transwell chamber, and filled the lower transwell chambers with 800 μL of DMEM containing 10% FBS as a chemoattractant. Cells were allowed to migrate for 48 h in the medium with liraglutide (100 nmol/L) and/or metformin (0.75 mmol/L). At the end of treatment, the non-migrated cells were removed from the upper surface by scraping with a wet cotton swab. The filter was fixed with formalin and stained with crystal violet. The number of cells in 6 random fields was counted under a microscope (200×).

### Western blot analysis

Cells were harvested and lysed with RIPA lysis buffer (Applygen Technologies Inc., Beijing, China) containing the protease inhibitor cocktail (Roche, Basel, Switzerland). Proteins were separated by 10% (*wt*/*vol*) SDS-PAGE and transferred to a nitrocellulose membrane. After incubating in the blocking solution (5% BSA in TBS-T), the membranes were incubated and probed with the primary antibodies (all at 1:1,000 dilution) overnight at 4°C, and subsequently incubated with IRDye 800CW-conjugated goat anti-rabbit IgG or goat anti-mouse IgG (both at 1:10,000 dilution) for 1 h. Protein bands were visualized with the Odyssey infrared imaging system (LI-COR Biosciences). GAPDH was used as the loading control.

### RT-PCR analysis

RNA of the cells was extracted with a Trizol kit (Life Technologies) and reversely transcribed to cDNA using a First Strand cDNA synthesis Kit (Fermentas, Burlington, ON, Canada). The cDNA was amplified by PCR using *Taq* Plus PCR Master Mix (Qiagen, Duesseldorf, Germany). The primer sequences specific for GLP-1 receptor (480 bp) were forward primer 5’-TCAAGGTCAACGGCTTATTAG-3’ and reverse primer 5’-TAACGTGTCCCTAGATGAACC-3’. The primer sequences specific for GAPDH (289 bp) were forward primer 5’-ACAGTCAGCCGCATCTTCTT-3’ and reverse primer 5’-CTGGAAGATGGTGATGGGAT-3’.

### Statistical analysis

Data are shown as means ± SD. Statistical differences between the groups were analyzed by one-way ANOVA followed by the post-hoc Tukey-Kramer test. *P*<0.05 was considered to be statistically significant. All analyses were performed using SPSS 20.0 for Windows (SPSS Japan Inc., Tokyo, Japan). Drug interaction was assessed as combination index (CI), which was calculated by CalcuSyn software program (Version 2.1, Biosoft, Cambridge, UK), and indicated as synergism (CI < 0.9), additivity (CI 0.9–1.1) or antagonism (CI > 1.1).

## Results

### Combined treatment with liraglutide and metformin is more effective than single treatment in inhibiting pancreatic cancer cell proliferation

**Fig 1:**
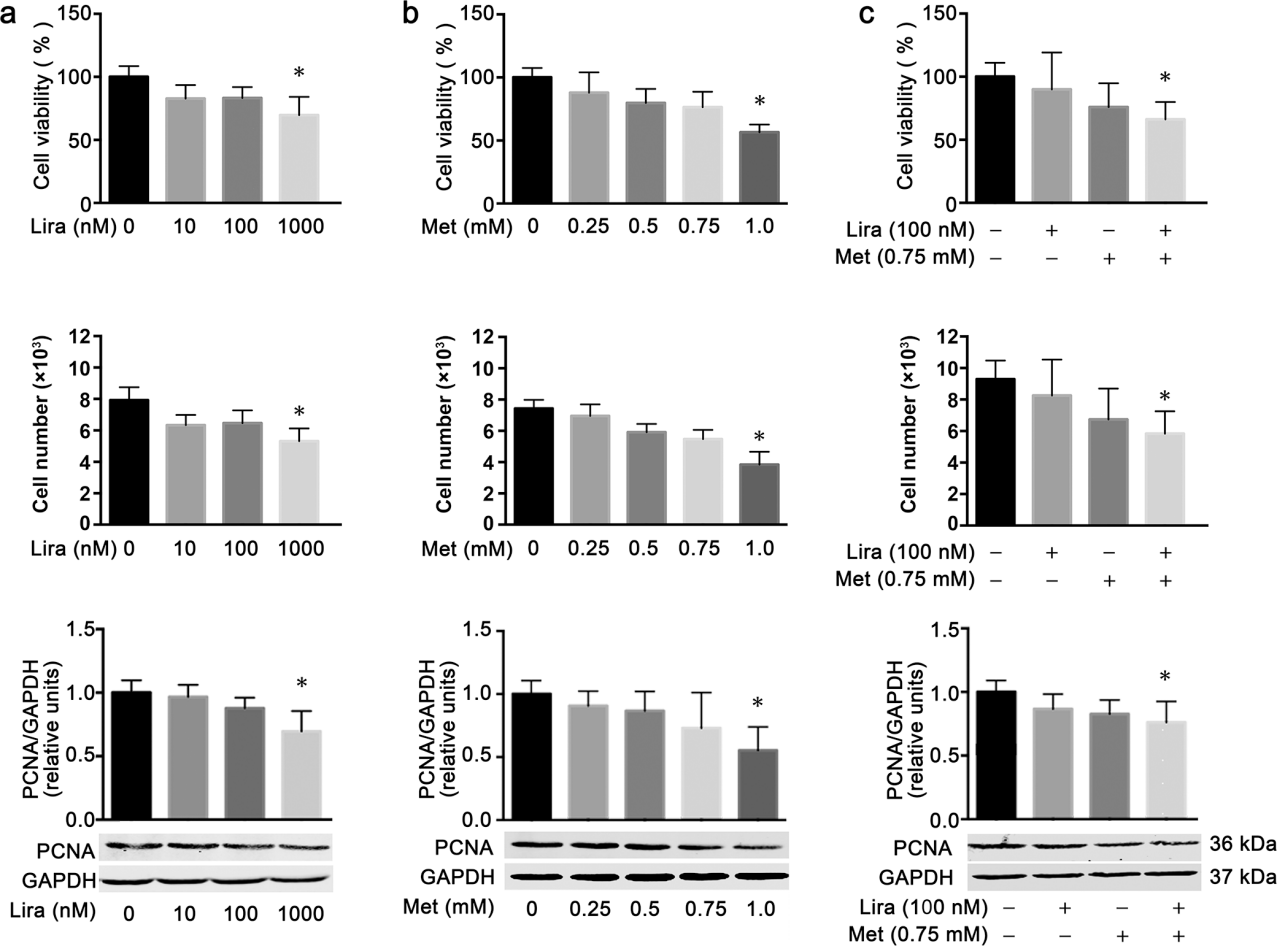
Combined effects of liraglutide and metformin on cell proliferation in human pancreatic cancer cells. MiaPaca-2 cells were incubated for 48 h with metformin (**a**) or liraglutide (**b**) alone or in combination (**c**) at the specified concentration. Cell viability (top) and cell numbers (middle) for each treatment group were measured using the CCK-8 assay, and the protein level of the cell proliferation marker PCNA was determined by western blot analysis (bottom). Data are shown as means ± SD. n = 4. *P<0.05 vs. control. Lira, liraglutide; Met, metformin; PCNA, proliferating cell nuclear antigen.

Compared with the untreated control, both liraglutide and metformin dose-dependently reduced cell viability and cell number in the human pancreatic cancer cell line MiaPaca-2 after 48 h of incubation. The inhibitory effects on cell viability and cell number were statistically significant only at the concentration of 1,000 nmol/L for liraglutide and 1.0 mmol/L for metformin (Fig 1A and 1B). Notably, the combination treatment of liraglutide (100 nmol/L) and metformin (0.75 mmol/L) also exhibited significant inhibitive effects on cell viability and cell number, which could not be achieved by the single treatment of these two drugs at the same concentration (Fig 1C). The CI value for the combined effects of liraglutide and metformin on cell viability was 0.712, which indicated the synergistic inhibitory effect of these two drugs on the growth of the pancreatic cancer cells. In agreement with these findings, both liraglutide and metformin could dose-dependently downregulate the protein level of PCNA, a cell proliferation marker, which was also significant only at the concentration of 1,000 nmol/L for liraglutide and 1.0 mmol/L for metformin (Fig 1A and 1B). Similarly, the combined treatment with liraglutide (100 nmol/L) and metformin (0.75 mmol/L) showed a significant downregulating effect on the PCNA protein level although the single treatment at the same concentration did not have such an effect (Fig 1C). These results indicated that combined treatment with liraglutide and metformin was more effective than the single treatment in inhibiting the proliferation of the human pancreatic cancer cells.

### Combined treatment with liraglutide and metformin causes reduced colony formation and cell cycle arrest in pancreatic cancer cells

**Fig 2:**
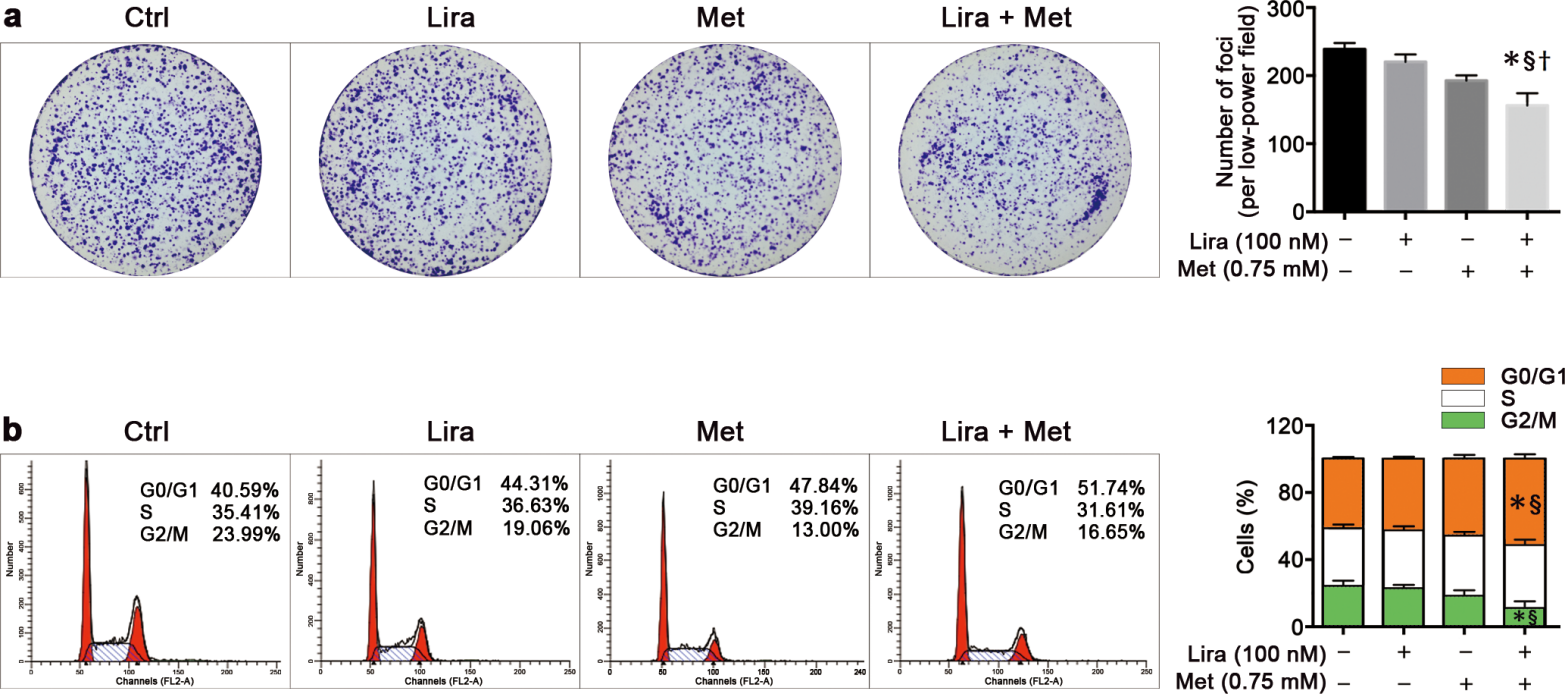
Combined effects of liraglutide and metformin on colony formation and cell cycle in human pancreatic cancer cells. MiaPaca-2 cells were treated with liraglutide (100 nmol/L) and/or metformin (0.75 mmol/L) for 8 days in the colony formation assay (**a**) or 96 h in cell cycle determination (**b**). The quantitative histograms of either colony formation or cell cycle distribution are shown in the right. Data are shown are means ± SD. n = 4. *P<0.05 vs. control; ^§^*P*<0.05 vs. liraglutide; ^†^*P*<0.05 vs. metformin. Ctrl, control; Lira, liraglutide; Met, metformin.

It is well established that there is a highly tumorigenic subpopulation in the pancreatic cancer cells, namely cancer stem cells, which play a more important role in cell mitosis and colony formation. To assess the combined effects of liraglutide and metformin on the pancreatic cancer stem cells, we evaluated the clonogenic survival by plating the cells at a very low density and counting the colony formation after 8 days. Results showed that MiaPaca-2 cells could not survive 8 days after treatment with metformin at a concentration of 3.0 mmol/L or more, and 1.0 mmol/L of metformin could significantly decrease the number of colonies after 8 days of treatment (data not shown). In addition, the inhibitory effect of liraglutide on clonogenic survival was significant only at a concentration of 1,000 nmol/L or more (data not shown). Either liraglutide (100 nmol/L) or metformin (0.75 mmol/L) alone could not significantly reduce the colony formation compared with the untreated control; however, when combined together at the same concentration of these two drugs, a significantly decreased colony formation in MiaPaca-2 cells was noticed when compared with the control or the single treatment with liraglutide or metformin (Fig 2A).

To further characterize the synergistic anti-proliferative effects of the combined treatment, cell cycle analysis was performed in MiaPaca-2 cells treated with liraglutide or metformin alone or in combination. Compared with the untreated control, liraglutide (100 nmol/L) in combination with metformin (0.75 mmol/L) could significantly cause cell cycle arrest at the G1 phase and consequently reduce the percentage of cells at the G2 phase. However, such effects could not be achieved by the single treatment of these two drugs at the same concentration. Besides, the combined effect of these two drugs was also significant when compared with the single treatment of liraglutide (Fig 2B).

### Combined treatment with liraglutide and metformin is more effective than single treatment in promoting pancreatic cancer cell apoptosis

**Fig 3:**
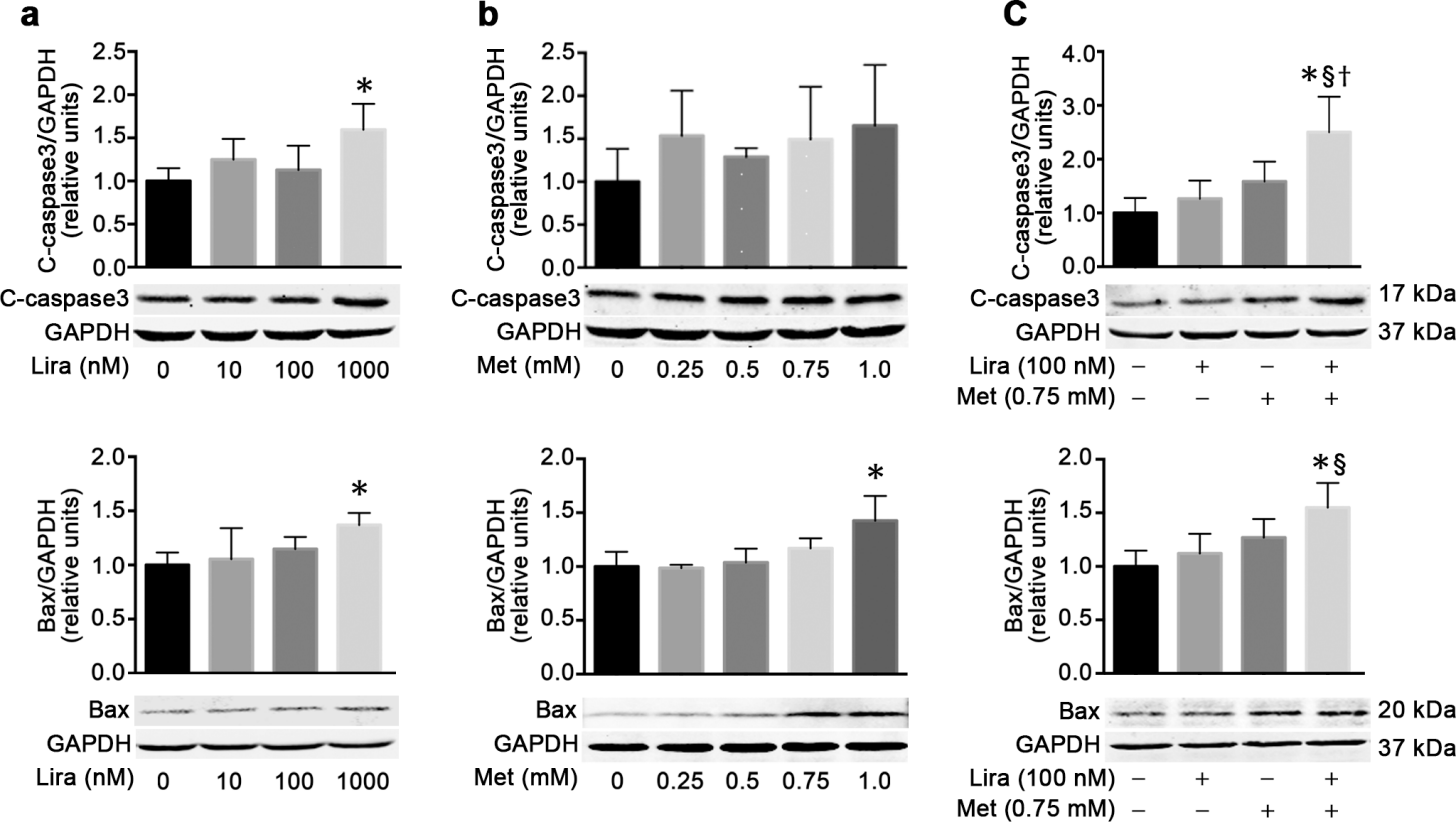
Combined effects of liraglutide and metformin on cell apoptosis in human pancreatic cancer cells. MiaPaca-2 cells were incubated for 48 h with liraglutide (**a**) or metformin (**b**) alone or in combination (**c**) at the specified concentrations. Protein levels of the pro-apoptotic marker cleaved caspase-3 (top) and Bax (bottom) were measured by western blot analysis. Data are shown as means ± SD. n = 4. *P<0.05 vs. control; ^§^*P*<0.05 vs. liraglutide; ^†^*P*<0.05 vs. metformin. Lira, liraglutide; Met, metformin.

In order to investigate whether the apoptosis process was involved in the inhibition of pancreatic cancer cell growth caused by treatment with liraglutide or metformin alone or in combination, the levels of the pro-apoptotic proteins Bax and cleaved caspase-3 were determined by western blot analysis. As expected, both liraglutide and metformin could dose-dependently upregulate the protein level of Bax in MiaPaca-2 cells, which was statistically significant only at the concentration of 1,000 nmol/L for liraglutide and 1.0 mmol/L for metformin. Moreover, the protein level of cleaved caspase-3 was significantly upregulated by 1,000 nmol/L liraglutide but not 1.0 mmol/L metformin (Fig 3A and 3B). Notably, the combined treatment with liraglutide (100 nmol/L) and metformin (0.75 mmol/L) could significantly upregulate the protein levels of both Bax and cleaved caspase-3 in MiaPaca-2 cells, although the single treatment of these two drugs at the same concentration did not have such effects (Fig 3C). These results indicated that combined treatment with liraglutide and metformin was more effective than their single treatment in promoting the human pancreatic cancer cell apoptosis.

### Combined treatment with liraglutide and metformin inhibits cell migration in pancreatic cancer cells

**Fig 4:**
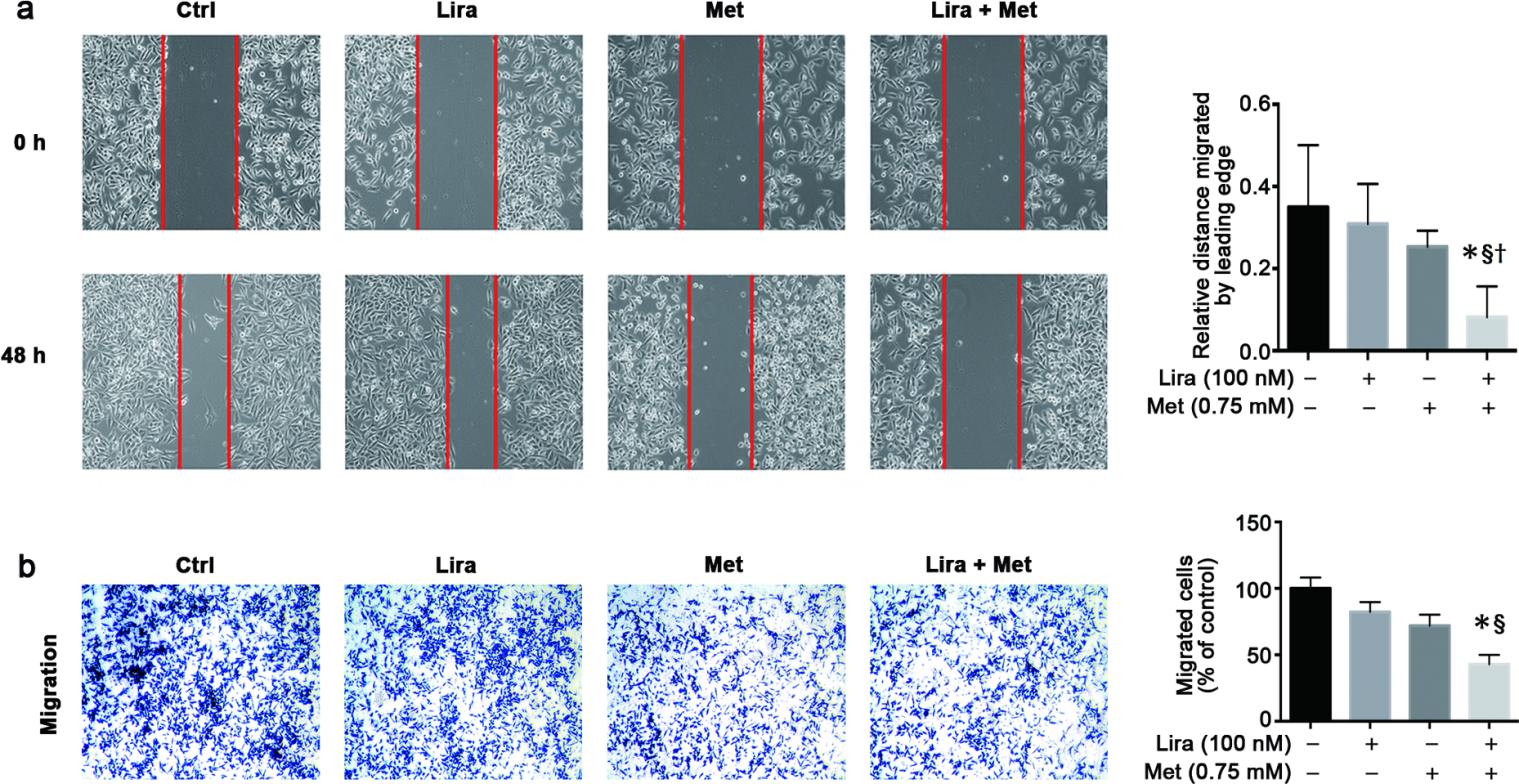
Combined effects of liraglutide and metformin on cell migration in human pancreatic cancer cells. MiaPaca-2 cells were treated with liraglutide (100 nmol/L) and/or metformin (0.75 mmol/L) for 48 h. Wound healing assay (**a**) and transwell migration assay (**b**) were performed to analyze cell migration, as shown by either photographs (left) or histograms (right). Data are shown as means ± SD. n = 4. *P<0.05 vs. control; ^§^*P*<0.05 vs. liraglutide; ^†^*P*<0.05 vs. metformin. Ctrl, control; Lira, liraglutide; Met, metformin.

The combined effect of liraglutide and metformin on the pancreatic cancer cell motility was determined by both wound-healing and transwell migration assays. In the wound-healing assay, either liraglutide (100 nmol/L) or metformin (0.75 mmol/L) could but did not significantly decrease the migration distance of MiaPaca-2 cells after 48 h of intervention; however, such an effect became significant when the combined treatment was given (Fig 4A). In the transwell migration assay, the greatest number of cells that migrated across the microporous filtering membrane was found in the control group. Treatment with either liraglutide (100 nmol/L) or metformin (0.75 mmol/L) alone had a tendency to decrease the number of cells that migrated across the membrane. Notably, the combined treatment of liraglutide and metformin further inhibited cell migration in the transwell migration assay, with a significantly lower number of the migrated cells compared with that in the untreated control and in the single treatment of liraglutide (Fig 4B).

### Combined treatment with liraglutide and metformin activates AMPK signaling more potently than single treatment in pancreatic cancer cells

**Fig 5:**
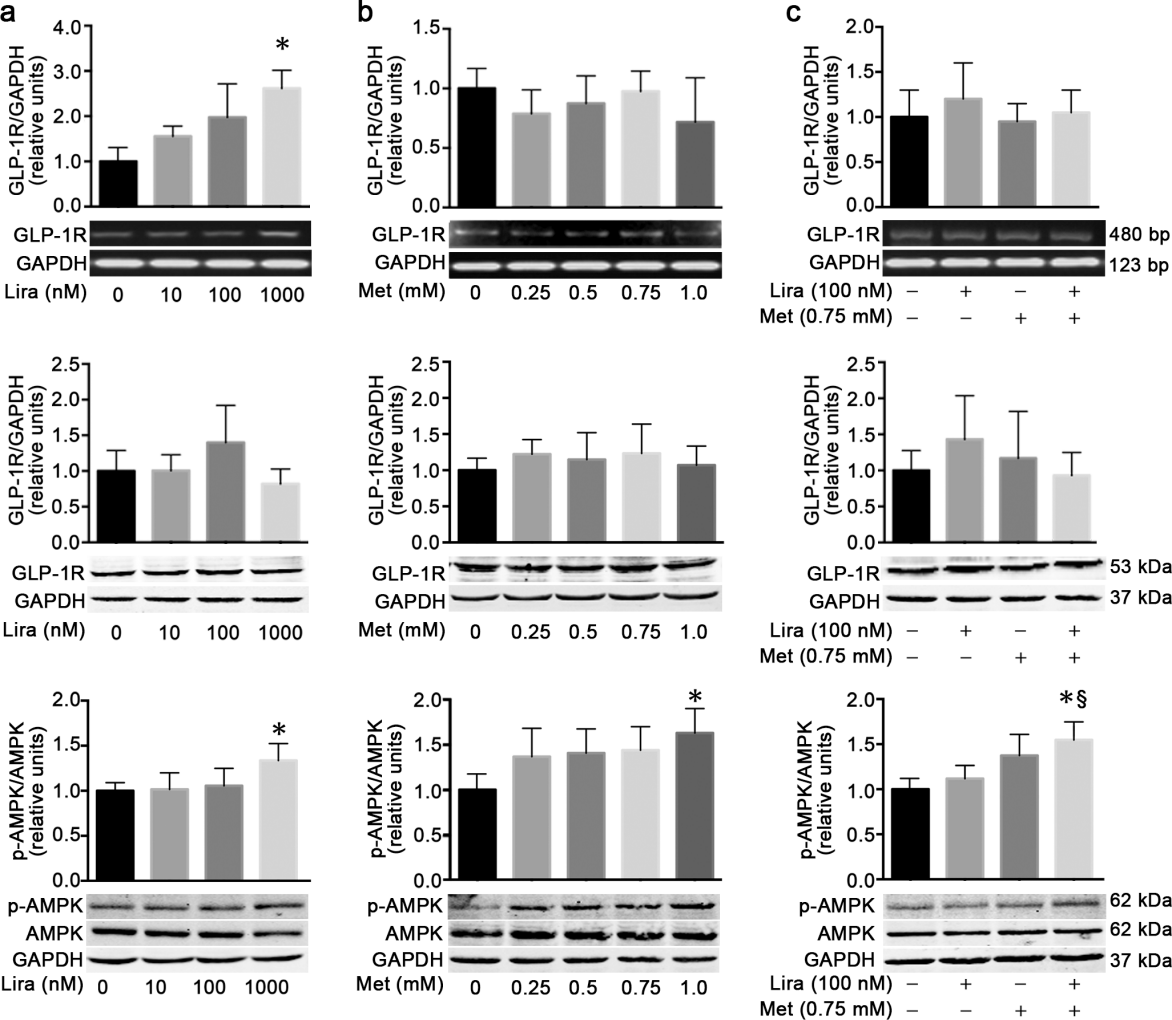
AMPK phosphorylation but not GLP-1 receptor level is upregulated by combination treatment with liraglutide and metformin in human pancreatic cancer cells. MiaPaca-2 cells were incubated for 48 h with liraglutide (**a**) or metformin (**b**) alone or in combination (**c**) at the specified concentrations. The levels of GLP-1 receptor (GLP-1R) mRNA (top) and protein (middle), and the protein level of p-AMPK (bottom) were measured by RT-PCR and/or western blot analyses. Data are shown as means ± SD. n = 4. *P<0.05 vs. control; ^§^*P*<0.05 vs. liraglutide. Lira, liraglutide; Met, metformin; GLP-1R, glucagon-like peptide-1 receptor; AMPK, AMP-activated protein kinase; p-AMPK, phosphorylated AMPK.

It is well known that liraglutide and metformin can exert their anti-diabetic effects through activation of GLP-1 receptor and AMPK signaling, respectively. To clarify whether GLP-1 receptor or AMPK signaling might also be involved in the synergistic anti-tumor effect of combined treatment with liraglutide and metformin on the pancreatic cancer cells, we determined the levels of GLP-1 receptor and p-AMPK using RT-PCR and/or western blot analyses in MiaPaca-2 cells treated with liraglutide or metformin alone or in combination. Although liraglutide (1,000 nmol/L) could significantly upregulate the mRNA expression level of GLP-1 receptor in MiaPaca-2 cells, the protein level of GLP-1 receptor was unchanged by the liraglutide treatment (Fig 5A). Single treatment with different concentrations of metformin failed to increase the mRNA and protein levels of GLP-1 receptor in MiaPaca-2 cells (Fig 5B). Moreover, combination treatment with metformin (0.75 mmol/L) and liraglutide (100 nmol/L) did not alter the mRNA and protein levels of GLP-1 receptor (Fig 5C).

As expected, metformin could dose-dependently upregulate the protein level of p-AMPK in MiaPaca-2 cells. The effect was statistically significant only at the concentration of 1.0 mmol/L metformin compared with the untreated control (Fig 5B). Interestingly, the protein level of p-AMPK also significantly increased after liraglutide treatment at the concentration of 1,000 nmol/L, although such an effect was not significant when the cells were treated with liraglutide at lower concentration (Fig 5A). Importantly, combination treatment with metformin (0.75 mmol/L) and liraglutide (100 nmol/L) induced a significant upregulation of p-AMPK protein level in MiaPaca-2 cells, which could not be achieved by the single treatment of these two drugs at the same concentration (Fig 5C).

## Discussion

Pancreatic cancer ranks as the fourth leading cause of cancer-related deaths [1]. There are limited effective treatments for patient with pancreatic cancer. Therefore, this disease has placed a great burden on public health [1]. Due to a high coexisting rate of diabetes and pancreatic cancer [2,4], it is quite common that patients with pancreatic cancer receive anti-hyperglycemic treatments at the same time as chemotherapy. Thus, it is of great importance to clarify the effects of the widely-prescribed anti-diabetic agents on the biological behaviors of pancreatic cancer cells.

It has been shown that metformin, a first-line anti-diabetic agent, has broad anti-tumor effects in numerous studies [17–22]. Patients with diabetes mellitus who were treated with metformin had lower incidences of breast cancer, lung cancer, colorectal cancer, hepatocellular carcinoma and pancreatic cancer than those in patients without metformin treatment in the observational studies [18]. Likewise, meta-analysis also showed that patients with diabetes mellitus who received metformin treatment had a 37% lower risk of developing pancreatic cancer and that metformin use was associated with better survival outcomes in patients with pancreatic cancer [17,18]. In addition, metformin attenuated the growth of human pancreatic cancer cells in a mouse xenograft model *in vivo* [20], and caused cell cycle arrest, inhibited cell migration and invasion, and exhibited anti-proliferative and pro-apoptotic effects on the pancreatic cancer cells *in vitro* [21,22]. Furthermore, metformin has the ability to enhance the effects of chemotherapy and radiotherapy and has been tested as an adjunctive therapy in treating patients with pancreatic cancer in dozens of clinical studies [6,23]. In accordance with those above observations, we found that 1.0 mmol/L metformin could significantly decrease cell viability, downregulate the protein level of the cell proliferation marker PCNA and upregulate the protein level of the pro-apoptotic marker Bax in the human pancreatic cancer cell line MiaPaca-2. These results suggested that metformin could inhibit tumor cell growth in the pancreatic cancer cells. It should be noteworthy that in the present study, metformin exhibited anti-tumor effects at the relatively low concentration of 1 mmol/L (typically 5–30 mmol/L in the other studies) [24], the reason for this might be probably that we cultured MiaPaca-2 cells in DMEM containing glucose at 4.5 mmol/L instead of 25 mmol/L used in most other studies. We chose the low-glucose media because glucose at 4.5 mmol/L might be much closer to the human physiological condition and because high glucose concentration itself could alleviate the anti-tumor effects of metformin [25].

Compared to a great deal of thrilling evidence on association of metformin treatment with risk of pancreatic cancer, the relationship between GLP-1 receptor agonists and pancreatic cancer risk is still controversial. Evaluation of the Food and Drug Administration Adverse Event Reporting System database showed a 10- and 2-fold increased risk of pancreatitis and pancreatic cancer respectively in patients treated with the GLP-1 receptor agonist exenatide in comparison to those receiving other anti-diabetic drugs, which raised a pancreatic safety concern about GLP-1-based therapies [26]. In that observational study, however, there was reporting bias existed obviously. Moreover, the level of body weight and the severity of diabetes, which were both associated with risk for pancreatitis and pancreatic cancer, were not well matched between the GLP-1 receptor agonist and control groups [27]. Such defects could also be found in the other observational or cohort studies [28]. In three large-scale, multicentre, randomized, double-blind, placebo-controlled cardiovascular outcome trials, patients who received GLP-1 receptor agonist (lixisenatide [29], liraglutide [10] or semaglutide [11]) treatment showed no significant increase in the incidence of either pancreatitis or pancreatic cancer compared with the well-matched control group, although serum levels of lipase and amylase were modestly elevated in the GLP-1 receptor agonist group. Likewise, conflicting results were also reported in laboratory studies investigating whether or not GLP-1 receptor agonists caused inflammation in pancreatic tissue and promoted proliferation in pancreatic cancer cells [7,30,31]. Nevertheless, it is worthy to note that GLP-1 receptor agonists exhibit anti-tumor effects in breast [32] and colon [33] cancer cells. Interestingly, our previous studies showed that liraglutide could dose- and time-dependently exert anti-proliferative and pro-apoptotic effects on the two human pancreatic cancer cell lines (MiaPaca-2 and PANC-1) *in vitro*, and retard the tumor growth of the human pancreatic cancer cells in mouse xenograft model *in vivo* [12,13]. In line with the observations from our previous studies, this study also showed that liraglutide could dose-dependently decrease cell viability and cell number, downregulate the protein level of the cell proliferation marker PCNA, and upregulate the protein levels of the pro-apoptotic markers Bax and cleaved caspase-3 in the human pancreatic cancer cell line MiaPaca-2.

As mentioned above, either liraglutide or metformin alone shows anti-tumor effects in the pancreatic cancer cells. However, it is not clear whether the combined treatment of these two drugs has an additive or synergistic effect on the pancreatic cancer cells. In the present study, we found for the first time that combination treatment with liraglutide and metformin synergistically inhibited cell growth (as shown by reducing cell viability, cell number, colony formation and PCNA protein level), promoted cell apoptosis (characterized by upregulating the protein levels of Bax and cleaved caspase-3), and suppressed cell migration (as indicated by both wound-healing and transwell migration assays) in the human pancreatic cancer cell line MiaPaca-2. Furthermore, it was previously reported that metformin alone resulted in G1 arrest [21] and liraglutide alone caused S/G2 arrest [12] in cell cycle distribution analysis. In our study, however, metformin in combination with liraglutide could cause G1 arrest with a higher percentage of cells at the G0-G1 phase, a lower percentage of cells at the G2 phase, and no significant change of cells at the S phase.

The mechanism of anti-tumor effects of metformin or liraglutide alone has been elucidated in several previous studies [12,13,24,34]. Apart from attenuating hyperinsulinemia or metabolic disorders *in vivo* and thus inhibiting insulin- or metabolite-dependent proliferative effects by both metformin and liraglutide [35,36], these two drugs also have direct anti-tumor effects in the *in vitro* studies [13,21,22,32]. Metformin can inhibit cancer cell growth through influencing various signaling pathways, of which LKB1 (liver kinase B1)/AMPK signaling pathway has been extensively studied [34]. In the present study, we demonstrated that metformin dose-dependently increased AMPK phosphorylation, thus activating the AMPK signaling pathway in MiaPaca-2 cells. Moreover, our previous studies showed that liraglutide inhibited the pancreatic cancer cell growth, migration and invasion through activation of cAMP production and consequent inhibition of the PI3K/Akt and ERK1/2 signaling pathways in a GLP-1 receptor-dependent manner [12,13]. So far, there is no report that investigates the signaling mechanism for the interactive effects of liraglutide and metformin in the pancreatic cancer cells. Notably, it has been suggested that in other tissue cell types, these two drugs may influence one another’s signaling pathway both *in vivo* and *in vitro*. For instance, metformin could increase the level of plasma active GLP-1 through stimulating GLP-1 secretion from intestinal L cells [37] or inhibiting GLP-1 degradation by dipeptidyl peptidase-4 [38], and upregulate the level of GLP-1 receptor in pancreatic beta [39] and endothelial cells [16]. On the other hand, liraglutide exerts a protective effect on endothelial function via activation of AMPK signaling pathway [40], which is analogous to the mechanism of metformin action in endothelial cells. Besides, combination of metformin and liraglutide has synergistic protective effects on endothelial function by increasing the expression of GLP-1 receptor and the activation of AMPK signaling [16]. Based on the above observations, we hypothesized that the synergistic anti-tumor effects of liraglutide and metformin might resulted from their interactive actions on GLP-1 receptor and/or AMPK signaling activation in the pancreatic cancer cells. In the present study, although we found with no surprise that liraglutide could upregulate GLP-1 receptor mRNA expression and metformin could increase AMPK phosphorylation dose-dependently in MiaPaca-2 cells, no upregulation of GLP-1 receptor level by metformin was observed. Interestingly, the protein level of p-AMPK significantly increased after liraglutide treatment, which is in accordance with the findings observed in endothelial cells [40]. Importantly, combination treatment with metformin (0.75 mmol/L) and liraglutide (100 nmol/L) significantly stimulated AMPK phosphorylation in MiaPaca-2 cells, although the single treatment did not have such an effect. These results suggested that the combined treatment with liraglutide and metformin could activate AMPK signaling more potently than their single treatment, which might partly contribute to their synergistic anti-tumor effect in the pancreatic cancer cells.

Our study has several limitations. First, our study was focused on the combined effects of liraglutide and metformin on the biological behaviors of the human pancreatic cancer cells, and the mechanism study was quite insufficient. Thus, further studies should be needed to clarify the underlying molecular mechanism of the combined effects in more detail. Second, we only used one human pancreatic cancer cell line in this study because MiaPaca-2 was more sensitive to liraglutide treatment than PANC-1, another human pancreatic cancer cell line, according to our previous studies [12,13]. However, the results should be verified in other pancreatic cancer cell lines. Third, the synergistic anti-tumor effect of liraglutide and metformin was not recapitulated *in vivo*, which should be testified in an animal model of human pancreatic cancer cell xenografts in the future study.

In summary, we show for the first time that the combination treatment with liraglutide and metformin can more effectively attenuate cell proliferation, promote cell cycle arrest and cell apoptosis, and inhibit cell migration than the single treatment in the human pancreatic cancer cell line MiaPaca-2. Moreover, the combined treatment with liraglutide and metformin can activate AMPK signaling more potently than their single treatment, which may account for the synergistic anti-tumor effect of this combination treatment. This study may provide a novel clue for pancreatic cancer treatment and support the rationale of using metformin in combination with liraglutide as a treatment option for patients with type 2 diabetes and pancreatic cancer.

## Supporting information

S1 Fig Combined effects of liraglutide and metformin on cell apoptosis in human pancreatic cancer cells. MiaPaca-2 cells were incubated for 48 h with liraglutide (**a**) or metformin (**b**) alone or in combination (**c**) at the specified concentrations. Protein and mRNA levels of the anti-apoptotic marker Bcl-2 were measured by western blot and RT-PCR analysis. Data are shown as means ± SD. n = 4. Lira, liraglutide; Met, metformin.
(TIF)

S2 Fig Combined effects of liraglutide and metformin on cell invasion in human pancreatic cancer cells. MiaPaca-2 cells were treated with liraglutide (100 nmol/L) and/or metformin (0.75 mmol/L) for 48 h. Transwell invasion assay were performed to analyze cell invasion, as shown by either photographs (left) or histograms (right). Data are shown as means ± SD. n = 4. Ctrl, control; Lira, liraglutide; Met, metformin.
(TIF)
