# Supplementary figures and images for: Synergistic anti-tumor effects of liraglutide with metformin on pancreatic cancer cells
Source: PLoS One. 2018 Jun 13;13(6):e0198938. doi: 10.1371/journal.pone.0198938 (PMC5999272; doi:10.1371/journal.pone.0198938)

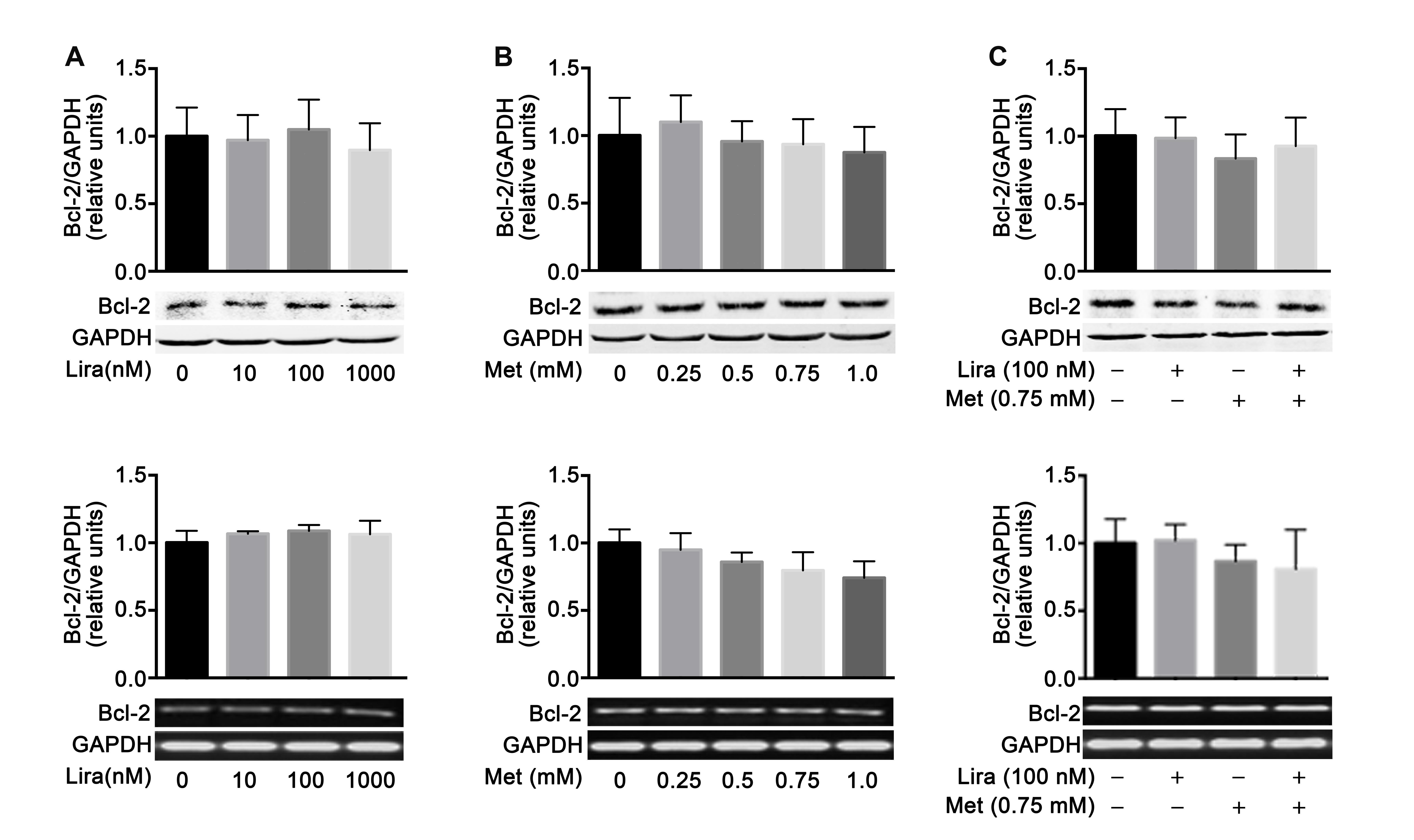

Supplement: S1 Fig — MiaPaca-2 cells were incubated for 48 h with liraglutide (a) or metformin (b) alone or in combination (c) at the specified concentrations. Protein and mRNA levels of the anti-apoptotic marker Bcl-2 were measured by western blot and RT-PCR analysis. Data are shown as means ± SD. n = 4. Lira, liraglutide; Met, metformin. (TIF) [file pone.0198938.s001.tif]

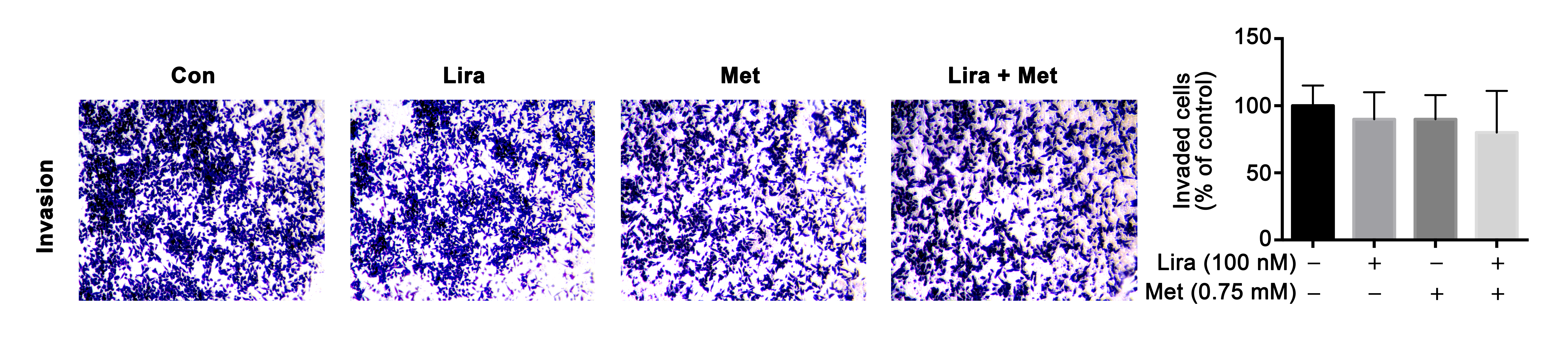

Supplement: S2 Fig — MiaPaca-2 cells were treated with liraglutide (100 nmol/L) and/or metformin (0.75 mmol/L) for 48 h. Transwell invasion assay were performed to analyze cell invasion, as shown by either photographs (left) or histograms (right). Data are shown as means ± SD. n = 4. Ctrl, control; Lira, liraglutide; Met, metformin. (TIF) [file pone.0198938.s002.tif]
